# Xanthogranulomatous Pyelonephritis With Extension to the Liver: A Case Report

**DOI:** 10.7759/cureus.42929

**Published:** 2023-08-04

**Authors:** Saad Bkiri, Fayçal Abbad, Mohammed Ghadouane

**Affiliations:** 1 Department of Urology, Cheikh Zaid International Hospital, Abulcasis International University, Rabat, MAR; 2 Department of Histopathology, Cheikh Zaid International Hospital, Abulcasis International University, Rabat, MAR

**Keywords:** nephrectomy, flexible ureterorenoscopy, liver abscess, xanthogranulomatous pyelonephritis, case report

## Abstract

Xanthogranulomatous pyelonephritis (XGP) is an uncommon condition representing 1% of all renal infections. XGP due to complicated pyelonephritis associated with hepatic abscess is an extremely rare complication and has rarely been reported in the literature. We report a rare case of a 54-year-old female with a history of diabetes and recurrent urinary tract infections (UTI) who presented with acute right flank pain and fever which had been ongoing for four days. CT scan showed multiple bilateral obstructive nephrolithiasis associated with a liver abscess. Given the patient’s high risk of nephron loss, a bilateral renal and liver abscess drainage followed by a two-stage flexible ureterorenoscopy (FURS) was performed. One week later, a CT scan showed a typical radiological aspect of XGP on the right kidney invading the liver. She urgently underwent a right nephrectomy with an uneventful outcome. In conclusion, the diagnosis of XGP should be considered in the presence of complicated pyelonephritis associated with hepatic abscess.

## Introduction

Xanthogranulomatous pyelonephritis (XGP) is a chronic inflammatory condition characterized by destructive granulomatous inflammation in the renal parenchyma [1]. It is named after the yellowish color (Xantho) observed during gross pathology and the presence of granulomatous reaction on histological examination [2]. XGP typically occurs as a result of chronic obstruction and suppuration, leading to severe inflammation in the renal parenchyma. The symptoms, signs, and laboratory findings associated with XGP are not specific, and a CT scan is considered the most accurate imaging technique to orient the diagnosis [3]. The final diagnosis of XGP is based on an anatomopathological study [2]. In our case report, we document an infrequent instance of XGP characterized by liver invasion, while delving into the details of our therapeutic approach.

## Case presentation

A 54-year-old female presented to our department with acute right-sided lower back pain irradiating to the right hypochondrium associated with fever, nausea, and vomiting for four days. The patient had hypertension, diabetes on medication, and a history of recurrent urinary tract infections (UTIs). The initial general examination revealed an asthenic patient who was febrile (temperature 38.9°C), conscious, and hemodynamically stable (blood pressure 12/84 mmHg). The abdominal exam revealed intense pain in the right flank.

The laboratory tests showed the following: anemia with hemoglobin level 9.1 g/dl, leukocytosis with a white blood cell count of 19.790/mm3, C-reactive protein (CRP) at 176.6 mg/L, hepatic cytolysis (alanine transaminase (ALT) 45 UI/l, aspartate transaminase (AST) 56 UI/l); cholestasis tests were normal. Renal failure was also noted with a creatinine level of 20.17 mg/L, and blood urea nitrogen (BUN) of 61 mg/dL with an estimated glomerular filtration rate of 31 ml/min/1.73m2 (Table 1).

**Table 1:** Table summarizing all the analyses conducted along with their normal values

The analysis	Patient's results	Normal values
Hemoglobin level	9.1 g/dL	12.3 gm/dL to 15.3 gm/dL
White blood cell	19.790/mm^3^	4.500–11.000/mm^3^
C-Reactive protein (CRP)	176.6 mg/L	<8mg/L
Alanine transaminase (ALT)	45 U/L	< 35 U/L
Aspartate transaminase (AST)	56 U/L	< 35 U/L
Creatinine level	20.17 mg/L	6 à 11 mg/L
Blood urea nitrogen (BUN):	61 mg/dL	7-20mg/dL

**Figure 1:**
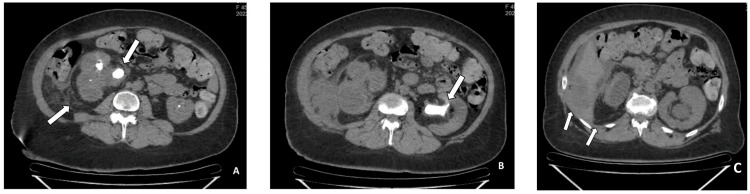
(CT scan demonstrating A) right hydronephrosis due to an obstructive stone with perirenal fat infiltration, (B) left hydronephrosis due to obstructive pyelo-ureteral junction (PUJ) stone, (C) hepatic abscess of segment IV extending into the perihepatic region and the adjacent retroperitoneal space

The urine culture revealed the presence of Escherichia coli with sensitivity to ciprofloxacin and ceftriaxone. The abdominal-pelvic CT scan revealed bilateral hydronephrosis due to obstructive urolithiasis. The right kidney demonstrated enlargement (137 x 81 x 63 mm) with lobulated contours and significant pyelo-calyceal dilatation (renal pelvis: 36 mm) upstream of an impacted stone at the pyelo-ureteral junction measuring 20 x 16 mm/and a density of 1255 Hounsfield units (HU). Notably, the ureter exhibited ectasia throughout its entire length, extending up to the ureteral orifice with a maximum diameter of 13 mm, without any visible obstruction. The left kidney also showed enlargement (144 x 82 x 69 mm) with finely notched contours and exhibited pyelo-calyceal dilatation (renal pelvis: 24 mm, corticomedullary medio-renal index: 12 mm) caused by a lower coralliform pyelocaliceal stone measuring 33 x 14 mm/1145 HU. A liver abscess (4 cm) was also noted in segment IV with an extension to the adjacent retroperitoneal space (Figure 1).

An urgent multidisciplinary consultation meeting was held to review the patient's case, and it was decided that a conservative approach would be taken initially due to the patient's high risk of nephron damage. The first step was to immediately perform bilateral kidney drainage, followed by a two-stage flexible ureterorenoscopy (FURS) for each side in a subsequent phase.

The liver abscess was echo-guided and drained. An urgent bilateral renal drainage was carried out immediately. Samples of urine and pus were collected from the renal pelvis and the liver abscess confirming the presence of Escherichia coli sensitive to ceftriaxone and ciprofloxacin. The patient received intravenous ceftriaxone 1 g 2x/day and ciprofloxacin 200 mg/day for 21 days. There was a two-week gap between the insertion of the JJ stent (Cook Urological, IN, USA) and the initial left-sided FURS. The second left FURS session was scheduled two weeks after the first one.

**Figure 2:**
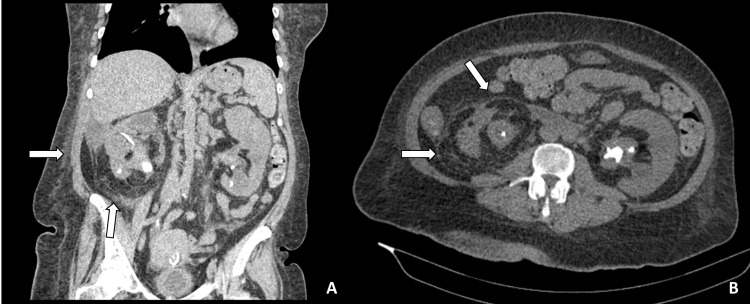
CT scan - (A) coronal section (B) transverse section showing the destruction of the right renal parenchyma with the extent of infiltration into the perirenal fat and retroperitoneal tissue

The complete fragmentation of the calculi was performed with a reusable ureterorenoscope, Holmium: Yag Laser (OmniPulse, Trimedyne, Irvine, CA, USA) and a 270nm fiber. A JJ stent was placed post-operatively after each FURS session. The operative duration of each FURS session did not exceed 80 minutes. The FURS for the right side were arranged to take place two weeks later. Unfortunately, the patient was subsequently admitted to the emergency department one week after her second left FURS due to an acute general condition decline associated with severe lower right back pain. After hemodynamic stabilization, an urgent CT scan was carried out revealing a renal architectural distortion, obliterated renal parenchyma with minimal corticomedullary differentiation, accompanied by extensive infiltration of peri-para-renal fat and Morison’s pouch space, in addition to a 20 x 16 mm obstructive kidney stone with a density of 1255HU compatible with a radiological aspect of XGP (Figure 2).

**Figure 3:**
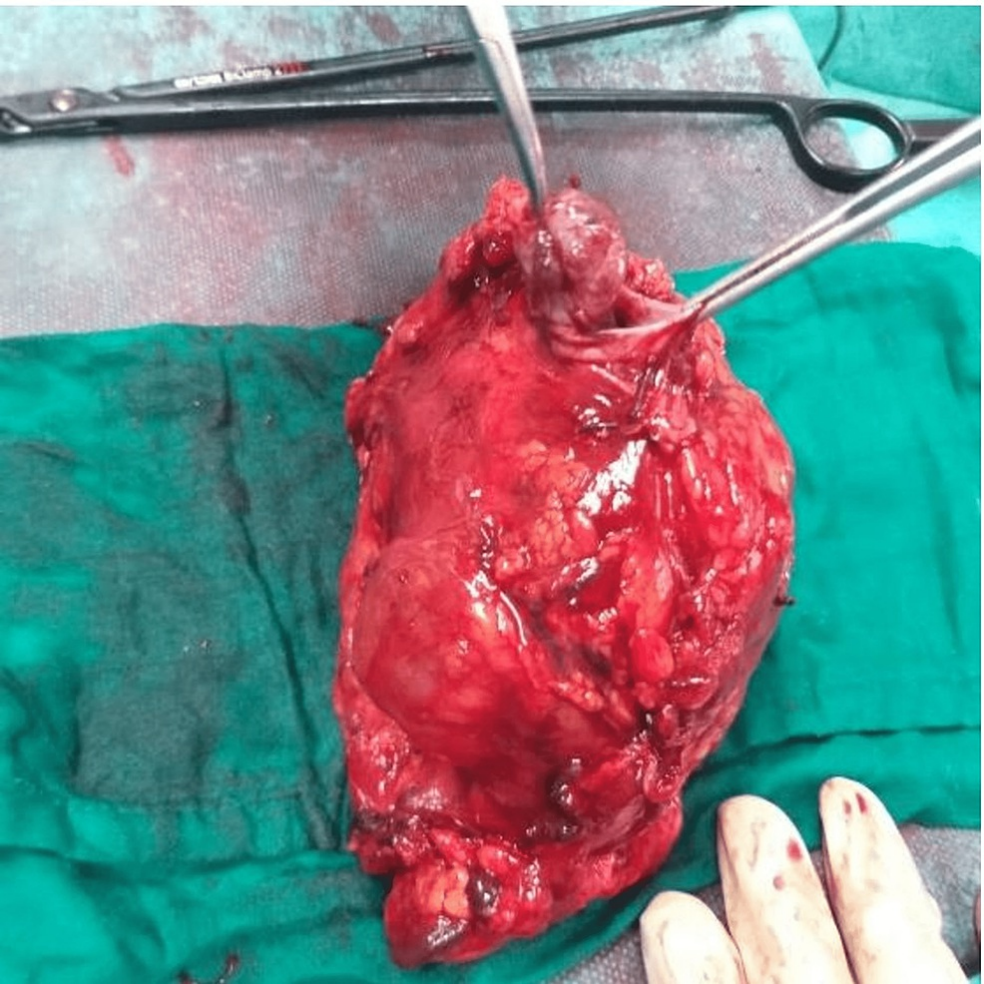
Macroscopic aspect of the removed kidney specimen

The patient was admitted to the operating room for a right total nephrectomy through open surgery (Figure 3).

The surgical operation was very challenging, especially during the dissection due to the inflammation of all the adjacent tissues. The operative duration was approximately two hours and 10 minutes with insignificant blood loss during surgery.

There were no complications during or after the surgery. A good clinical and biological evolution was noted. The antibiotic treatment was maintained for a period of one month following the surgery: ceftriaxone 200 mg, 1 pill 2x/day, and ciprofloxacin 500 mg, 1 pill 2x/day, orally, for one month. The plasma creatinine level decreased to 15.2 mg/L.

**Figure 4:**
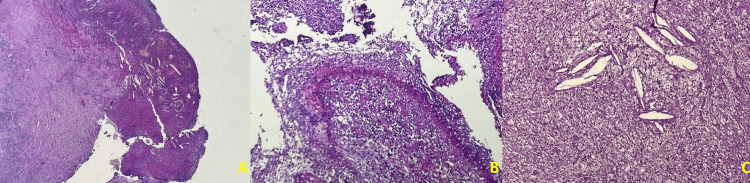
Histological image confirming XGP (A) x10 renal parenchyma completely remodeled and destroyed by the same inflammatory infiltrate; (B) x20 fibro-adipose involution remodeled by a cell population rich in foamy histiocytes; (C) x20 cholesterol-rich artifacts surrounded by a polymorphic inflammatory population rich in foamy histiocytes.

Histopathological examination confirmed the XGP diagnosis with a renal parenchyma completely remodeled and destroyed by the histological inflammatory infiltrate, as well as a fibro-adipose involution remodeled by a cellular population rich in foamy histiocytes (Figure 4). The spectrophotometric study revealed calculi of weddellite (IIb).

## Discussion

XGP is an uncommon chronic infection that affects the renal parenchyma. In 1916, Schlagenhaufer initially named it 'Staphylomykosen' and identified Escherichia coli and Proteus mirabilis as the primary microorganisms responsible for causing it [4]. This illness is widespread among individuals, but it has a higher incidence in females after middle age [5].

Several risk factors have been described in the literature, the most common ones being obstructive uropathy, diabetes, obesity, recurrent UTI, and immunosuppression [2]. Our patient fits in this spectrum (54 years old, diabetic, obese, and recurrent UTI with bilateral nephrolithiasis).

XGP has always been considered to be the 'big imitator'. It’s very difficult to recognize it from other medical conditions like chronic pyelonephritis, pyonephrosis, tuberculosis, or renal cell carcinoma only by clinical symptoms, physical examination, or standard imaging [3]. Usually, the abnormality affects only one side; there is no significant difference in occurrence between the right and left kidneys. However, when XGP is bilateral, which is an infrequent occurrence, it tends to have a lethal outcome [6]. Even though the disease process impacts the entire kidney, there have been descriptions of focal forms, albeit they are extremely uncommon [7]. The XGP described in our case is unilateral and widespread to the liver. This situation can be explained by the dissemination of the inflammatory process from the kidney to the liver. This can happen due to either direct invasion of inflammation through contiguous structures or via a hematogenous route.

The most common clinical symptoms in XGP patients are flank pain, fever, weight loss macroscopic hematuria, palpable mass, and urinary tract symptoms [1]. The use of a CT scan is widely regarded as the most precise imaging modality to orient the diagnosis [3]. However, until today, it cannot be used to confirm it with complete certainty. Typically, the final diagnosis of XGP is made through pathological examination rather than clinical preoperative assessments [2]. The ultrasound is nonspecific, and often demonstrates hydronephrosis, hypoechoic area, and calculi, in addition to a notable reduction in thickness or complete absence of the renal parenchyma [1,3]. However, it’s still considered to be an operator-dependent examination. CT scan is the most valuable imaging technique for both localized and widespread presentations of XGP [3]. CT allows visualization of dilated calyces, nephrolithiasis, dimensions, and morphology kidney changes as well as any complications that may be linked to XGP. The most distinctive feature is the 'bear paw sign' which may be seen with both cortical atrophy and dilatation of the calyces [2]. The MRI can be an alternative if a CT scan is prohibited. It is essential to have a radiology expert present during multidisciplinary case discussions as they play a vital role in distinguishing between XGP and other potential differential diagnoses [3].

In our case, the first abdominopelvic CT scan performed could not definitively show direct radiological signs in favor of XGP that could lead to radical treatment in our patient who was considered to be at high nephron risk. It was only on the second CT scan, carried out after two months, that the radiologist was able to orient the diagnosis towards XGP.

If there is uncertainty regarding the diagnosis, a biopsy may be carried out. Two biopsies were performed by a specialized medical team on a suspected malignant focal mass in the kidney, with no nephrolithiasis affirming XGP [8]. Another instance involved a confirmed case of focal XGP which was diagnosed through renal biopsy: The patient responded favorably to medical treatment, and unnecessary nephrectomy was avoided [9].

In addition to the potential risk of invasion to nearby organs such as the duodenum, spleen, psoas muscle, colon, and large blood vessels, some patients may also have cutaneous fistulas or urinothorax [1,10]. The extension to the liver is a very rare entity, there have been only a few cases described in the literature.

Setting up a treatment plan before surgery can be difficult because it is often challenging to make a diagnosis before the operation. The therapeutic approach is determined by the extent of infiltration of the lesions. If the XGP is focal, the primary therapeutic approach involves intravenous anti-biotherapy alongside renal drainage [1]. In cases where there is evidence of unfavorable progression, total or partial nephrectomy may be suggested as a potential intervention [1]. However, if the XGP is diffuse it’s recommended to perform a large nephrectomy covered by intravenous antibiotics to prevent any septic complications [11]. XGP with liver invasion is a rare condition that poses diagnostic challenges due to its atypical features. Through our case report, we provide an in-depth analysis of an uncommon presentation and its management, thereby contributing to the medical literature. Managing XGP with liver extension requires a multidisciplinary approach, often involving a radiologist, general surgeon, nephrologist, and urologist.

## Conclusions

Nephrolithiasis, a common disease, can contribute to the development of XGP in high-risk populations. There are only a few cases described in the literature concerning XGP with liver invasion. This report raises awareness of the association between chronic nephrolithiasis and XGP associated with hepatic abscess, thereby aiming to improve diagnosis and guide patients toward appropriate management.
